# Molecular Cloning and Characterization of Two Genes Encoding Dihydroflavonol-4-Reductase from *Populus trichocarpa*

**DOI:** 10.1371/journal.pone.0030364

**Published:** 2012-02-17

**Authors:** Yan Huang, Jiqing Gou, Zhichun Jia, Li Yang, Yimin Sun, Xunyan Xiao, Feng Song, Keming Luo

**Affiliations:** 1 Key Laboratory of Eco-Environments of Three Gorges Reservoir Region, Ministry of Education, Institute of Resources Botany, School of Life Sciences, Southwest University, Chongqing, China; 2 Chongqing Key Laboratory of Transgenic Plant and Safety Control, Southwest University, Chongqing, China; 3 Forage Improvement Division, The Samuel Roberts Noble Foundation, Ardmore, Oklahoma, United States of America; University of New South Wales, Australia

## Abstract

Dihydroflavonol 4-reductase (DFR, EC 1.1.1.219) is a rate-limited enzyme in the biosynthesis of anthocyanins and condensed tannins (proanthocyanidins) that catalyzes the reduction of dihydroflavonols to leucoanthocyanins. In this study, two full-length transcripts encoding for *PtrDFR1* and *PtrDFR2* were isolated from *Populus trichocarpa*. Sequence alignment of the two PtrDFRs with other known DFRs reveals the homology of these genes. The expression profile of *PtrDFRs* was investigated in various tissues of *P. trichocarpa*. To determine their functions, two *PtrDFR*s were overexpressed in tobacco (*Nicotiana tabacum*) via *Agrobacterium*-mediated transformation. The associated color change in the flowers was observed in all *35S:PtrDFR1* lines, but not in *35S:PtrDFR2* lines. Compared to the wild-type control, a significantly higher accumulation of anthocyanins was detected in transgenic plants harboring the *PtrDFR1*. Furthermore, overexpressing *PtrDFR1* in Chinese white poplar (*P. tomentosa* Carr.) resulted in a higher accumulation of both anthocyanins and condensed tannins, whereas constitutively expressing *PtrDFR2* only improved condensed tannin accumulation, indicating the potential regulation of condensed tannins by *PtrDFR2* in the biosynthetic pathway in poplars.

## Introduction

Flavonoids, very important secondary metabolites, exist widely throughout the plant kingdom. Presently, over 8,000 different compounds of flavonoids have been identified, many of which are involved in several biological processes, such as pigmentation of flowers, protection against UV-B injury, defense against pathogens and pests, pollen viability, auxin transport regulation, etc. [1], [2]. Flavonoids are divided into several structural subclasses, including flavanones, isoflavonoids, anthocyanins, flavonols, catechins, flavones and proanthocyanidins (also called condensed tannins or CTs), and are abundant in fruits, leaves and flowers [3]. The biosynthetic pathways of flavonoids are well established in plants [1], [4], [5].

In the flavonoid biosynthetic pathway, dihydroflavonol-4-reductase (DFR, EC 1.1.1.219) is one of the rate-limited enzymes that catalyzes the stereospecific reduction of three dihydroflavonols (dihydrokaempferol, dihydroquercetin and dihydromyricetin) to leucoanthocyanidins (flavan-3,4-diols) using NADPH as a cofactor [2], [6], [7]. Leucoanthocyanidins, the precursors of the anthocyanin branch, are essential for the formation of CTs [8]. Previous studies demonstrated that deactivation of the DFR gene resulted in the loss of anthocyanins and CTs in mutants of barley and Arabidopsis [9], [10]. Due to their crucial role in the flavonoid pathway, various DFR genes have been isolated from different species, such as *A. thaliana*, maize (*Zea mays*), barley (*Hordeum vulgare*), trembling aspen (*Populus tremuloides*), *Medicago truncatula* and *Petunia hybrida* [8], [11]–[15]. Variable DFR genes were therefore found in various genomes, i.e., a single copy *DFR* is present in *A. thaliana*, barley, tomato (*Lycopersicon esculentum*), grape (*Vitis vinifera*), snapdragon (*Antirrhinum majus*) and rice (*Oryza sativa*), while multi-copy *DFR*s exist in *P. hybrida* (Line V30), *Ipomoea purpurea*, *I. nil* and *M. truncatula* [7], [8], [11], [16]–[18].

Poplar (*Populus* spp.), as a model plant in trees, is extensively used in studies of tree morphology, physiology, biochemistry, ecology, genetics and molecular biology [19], [20]. A wide range of genomic and genetic resources is now available in the species, including an EST database [21], genome sequence and annotation from P. trichocarpa [22]. Previously, it was found that two duplicated *PtrDFR* genes are present in the P. trichocarpa genome [23]. Furthermore, a DFR mRNA was isolated from *P. tremuloides*, and its expression induced wounded leaves in aspen [7]. Phytochemical assays revealed that CT concentrations were significantly increased in wounded leaves. It suggested that CT synthesis is an inducible defense response in trembling aspen. However, the DFRs' functions in the biosynthetic pathway of other flavonoids, such as anthocyanidins, remain unclear.

In this study, we isolated two full-length mRNAs of *PtrDFR1* and *PtrDFR2*, which encode DFR isoenzymes from *P*. *trichocarpa*. The expression profiles of *PtrDFR*s were investigated in various tissues of *P*. *trichocarpa*. Both *PtrDFR1* and *PtrDFR2* were overexpressed in tobacco (*Nicotiana tabacum* cv Xanthi) and Chinese white poplar (*P. trichocarpa* Carr.), and their potential biological functions in the flavonoid biosynthetic pathways were investigated.

## Materials and Methods

### Plant materials and bacterial strains

Poplar plants were grown in the greenhouse at 26°C under 14/10h photoperiod with supplemental light (reached 600 footcandles). Individual tissues, including leaf, stem, root and petiole, were harvested separately and frozen in liquid nitrogen until further processing. Transgenic tobacco (*N. tabacum* cv Xanthi) and *P*. *tomentosa* Carr. (clone 73) were grown under the same conditions.

*Escherichia coli* strain DH5α was used as the recipient for transformation, genetic manipulation and production of plasmid DNA for sequencing. *Agrobacterium tumefaciens* strain LBA4404 was used as the disarmed vector for the transformation of tobacco and poplar.

### Isolation of total RNA and cloning of *PtrDFR* cDNAs from *P*. *trichocarpa*

Total RNA was isolated from frozen tissues of poplar (*P. trichocarpa*) plants using *RNeasy Plant* Mini *Kit* (Qiagen, Germany) following the manufacturer's instructions with a modification as we reported previously [24]. The leaves and petioles were excised from stems and included the fourth (young) and fifth (old) internodes from the top of the stems (1 m height). Samples from at least five stems were pooled for analysis. First-strand cDNA was synthesized from 2 µg of DNase-treated RNA with RT-AMV transcriptase (TaKaRa, Dalian, China) in a total volume of 20 µL by using oligo (dT) at 42°C for 30 min. The ORF of *PtrDFR*s was obtained by reverse transcription-PCR (RT-PCR) with 2 µl of cDNA from the roots, by using PtrDFR1-F: 5′-GC*GAGCTC*GATGGGAACAGAAGCTGAAAC-3′ (as forward primer, the *Sac*I site is in italics) and PtrDFR1-R: 5′-GC*GGATCC*GTGGAACAATCAGGACGCAG-3′ (as reverse primer, the *Bam*HI site is in italics) for *PtrDFR1*, and using PtrDFR2-F: 5′-GC*GAGCTC*CATGGGAGTAGAAGTTGAAAC-3′ (as forward primer, the *Sac*I site is in italics) and PtrDFR2-R: 5′-GC*GGATCC*AAACTAAAGGGCCTCAGAATC-3′ (as reverse primer, the *Bam*HI site is in italics) for *PtrDFR2*. The PCR reaction was carried out with Pfu DNA polymerase (TaKaRa, Dalian, China) in a total volume of 50 µl at 94°C for 5 min; 35 cycles of 94°C for 30 s; 56°C for 30 s; and 72°C for 2 min; followed by a final extension of 72°C for 7 min. The A tail was added to the PCR product, which was then cloned into pGEM-T easy vector according to the manufacturer's instructions (Promega), and the correct reading frame of the resulting construct was confirmed by sequencing. The PCR product was cloned into the *Sac*I and *Bam*HI sites of the plant binary vector pBI121. The resulting vectors, p*35S:PtrDFR1* and p*35S:PtrDFR2*, with *PtrDFR* ORFs driven by the cauliflower mosaic virus 35S (CaMV 35S) promoter and the *NPTII* gene as a plant-selectable marker conferring kanamycin resistance were transferred into *A. tumefaciens* strain LBA4404 by the freeze-thaw method.

### Transformation of tobacco plants

For tobacco transformation, *Agrobacterium* strain LBA4404 containing the binary vector was incubated in liquid YEP medium supplemented with 200 µM acetone-syringone [25], [26] at 28°C with constant shaking (200 rpm) until the culture reached an optimal density of approximately 0.6–0.8 at 600 nm. The *A. tumefaciens* culture was then diluted with an equal volume of liquid Murashige and Skoog medium [27]. Leaf discs from *N*. *tabacum* Xanthi were transformed as described previously [28]. Transformed plants were grown on Murashige and Skoog medium with 100 mg l^−1^ kanamycin under long day conditions (18/6 h photoperiod) at 25°C. Only one bud was picked from each explant to ensure independent transformants. Antibiotic-resistant plants were maintained as transgenic lines, and plantlets were transplanted to soil.

### Transformation of poplar plants

Transgenic Chinese white poplar (*P*. *tomentosa* Carr. clone 73) plants were generated by *Agrobacterium*-mediated transformation as described previously [29]. Recombinant *Agrobacterium* was used to infect poplar leaf discs, and putative transgenic plants were grown and selected on woody plant medium (WPM) [30] with 100 mg l^−1^ kanamycin. Rooted plantlets were acclimatized in pots at 25°C in a 16/8 h photoperiod and then transferred to the greenhouse for further studies.

### DNA extraction and PCR analysis

Genomic DNA was extracted from leaves (300 mg) of untransformed control and hygromycin-resistant plants using the modified CTAB extraction method as previously described [28]. To determine the presence of transgenes, putative transgenic plants were screened preliminarily by PCR analysis [31]. The following primers were designed for the *NPTII* gene – forward primer: 5′-AGGCTATTCGGCTATGACTGG-3′; reverse primer: 5′-TCGGGAGCGGCGATA CCGTA-3′. PCR conditions were 94°C for 3 min; 94°C for 30 s; 56°C for 30 s; and 72°C for 1 min–34 cycles in total. The PtrDFR1-F, PtrDFR1-R, PtrDFR2-F and PtrDFR2-R as described above were used for amplification of *PtrDFR1* and *PtrDFR2*, respectively. PCR amplification was performed by using a thermocycler (Eppendorf AG, Germany). Amplified DNA was loaded on 0.8% (w/v) agarose gel and visualized after ethidium bromide staining.

### RT-PCR analysis

To determine the presence of the transgenes in transgenic tobacco, total RNA was extracted from wild-type and transgenic plants using RNeasy Plant Mini Kit (Qiagen, Germany). For RT-PCR, DNase-treated RNA (2 µg) was then reverse transcribed in a total volume of 20 µl by using RT-AMV transcriptase (TaKaRa, Dalian, China) with oligo (dT) at 42°C for 30 min. Primers for *Actin*, which was used as an internal reference, were F: 5′-TGGACTCTGGTGATGGTGTC-3′ and R: 5′-CCTCCAATCCAAACACTGTA-3′. The number of cycles for each gene was varied to confirm that the PCR amplification was in the linear range as we described previously [32]. To detect *PtrDFR* expression, amplification was conducted for 26 cycles, with each cycle consisting of 94°C for 1 min; 58°C for 30 s; 72°C for 2 min; and finally 7 min at 72°C. Aliquots of individual PCR products were compared by agarose gel electrophoresis and visualized with ethidium bromide under UV light.

### Quantitative real-time PCR

Total RNA extracted from leaves, roots, stems and petioles of poplar plants at different developmental stages was treated with DNase I (TaKaRa, Dalian, China) according to the manufacturer's instructions. All RNA was purified and first-strand cDNA synthesized as described above. The reverse transcribed cDNA samples were used for real-time PCR, which was performed on a BioRad IQ5 real-time PCR detection system. The forward and reverse primers for *PtrDFR1* amplification were qtDFR1-F (5′-TACAATGTCCCTGCTAAGTTC-3′) and qtDFR1-R (5′-GTGGAACAATCAGGACGCAG-3′), and primers for *PtrDFR2* amplification were qtDFR2-F (5′-TACAGCTTGGAGGAAATGTTC-3′) and qtDFR2-R (5′-AAACTAAAGGGCCTCAGAATC-3′). The efficiency of these primers was investigated by applying primer melting curve analysis and gel electrophoresis; both results indicated that each primer pair gave a specific and unique PCR product. A *Populus Actin* gene, amplified with the primers Actin-F (5′-GTGCTTCTAAGTTCCGAACAGTGC-3′) and Actin-R (5′-GACTACCAAAGTGTCTGACCACCA-3′) giving a product of 180 bp, was used as a reference for loading normalization. Quantitative real-time PCR reaction and data analysis were performed as described by Tsai et al. [22] in a 20-µl reaction volume containing 10 µl of SYBR Green Master Mix reagent (TaKaRa, Dalian, China). Each experiment was performed in duplicate and with three biological replicates along with no-template controls. Statistical differences in expression between the mean values of the control and tested samples were analyzed by one-way analysis of variance (ANOVA) and the LSD test.

### Sequence comparisons and phylogenetic analysis

The nucleotide and deduced amino acid sequences were analyzed by DNAstar programs and DNAMAN software (Lynnon Corporation, USA). Amino acid sequence alignments were performed with DNAMAN software. The phylogenetic tree of DFRs was constructed by the neighbor-joining method for *P*. *trichocarpa* and other plants using DNAMAN software.

### Anthocyanin measurement

Anthocyanin quantification was performed as described by Pang et al. [33]. The poplar tissues were ground in liquid nitrogen and placed into 5 mL extraction buffer (an equal volume of methanol:0.1% HCl), sonicated for 1 h and then shaken in darkness for 4 h at 120 rpm. After centrifugation at 2,500 g for 10 min, 1 mL of water was added to 1 mL of extract, followed by the addition of 1 mL of chloroform to remove chlorophyll. The absorption of the extracts was measured spectrophotometrically at 530 nm. The amount of anthocyanins was reported as (*A*_530_) g^−1^ fresh weight (FW). The experiment was repeated three times for each treatment.

### Extraction and quantification of condensed tannins

Quantification of total condensed tannins from wild-type and transgenic poplar was performed using the vanillin-HCl method as described previously [34]–[36]. Leaf tissues were ground in liquid nitrogen and extracted with 5 mL of extraction solution (a 0.5% (v/v) vanillin solution in methanol containing 4% HCl). Following centrifugation at 2,500 g for 10 min, the residues were re-extracted twice, as above. Pooled supernatants were incubated for 20 min at room temperature. Samples, blanks and standards were read at 500 nm using a UV/Vis spectrophotometer, zeroing the spectrophotometer with deionized water. Blanks were subtracted from samples and condensed tannin content calculated as catechin equivalents. The concentration of condensed tannins was detected in triplicate.

## Results and Discussion

### Isolation and characterization of two DFR genes from *Populus trichocarpa*

**Figure 1:**
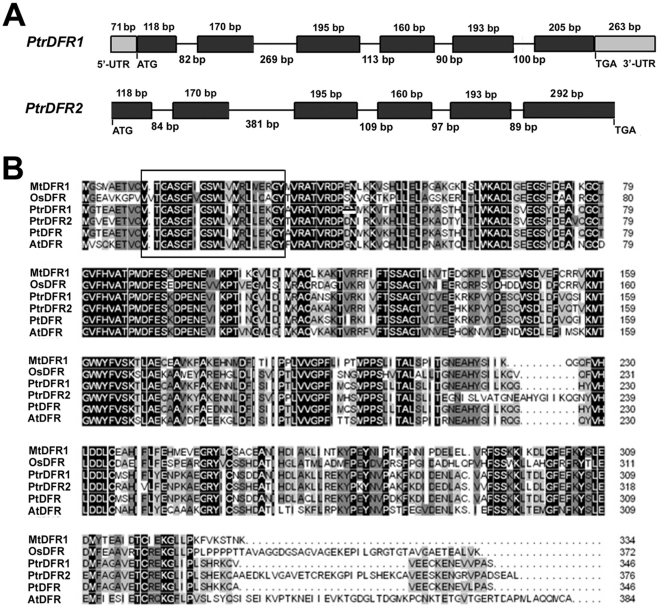
Nucleotide sequence analysis of *PtrDFR*s and their protein sequence alignments with other DFRs. (**A**) A schematic representation of the exon and intron organization of *PtrDFR*s. *PtrDFR1* consists of six exons (black boxes) and five introns (intervening line) with a 71-bp 5′ untranslated region (UTR; white box) and a 263-bp 3′ untranslated region. *PtrDFR2* also consists of six exons (black boxes) and five introns (intervening line). (**B**) The amino acid sequence and structure of PtrDFRs. The boxed region represents a putative NADPH binding domain at the N terminus of PtrDFR proteins. GenBank accession numbers are as follows (in parentheses): PtrDFR1 (XM_002300723), PtrDFR2 (XM_002307631), PtDFR (AY147903), AtDFR (AAA32783), MtDFR1 (AY389346), MtDFR1 (AY389347) and OsDFR (BAA36182).

The *Populus* genome was recently sequenced [21]. Based on the sequences deposited in the *Populus* genome database, bioinformatic analysis indicated that DFR was encoded by two genes [22] named *PtrDFR1* and *PtrDFR2* which consist of six exons and five introns (Figure 1A). The *PtrDFR1* cDNA contains 1,375 nucleotides, including a full-length open reading frame (ORF) encoding 346 amino acids, a 71-bp 5′ untranslated region (UTR) and a 263-bp 3′ UTR (GenBank Accession No. XM_002300723). The *PtrDFR2* cDNA contains a truncated ORF encoding 336 amino acids (GenBank Accession No. XM_002307631). Based on the alignment of the complete genomic sequence in the public database (JGI, http://genome.jgi-psf.org/cgi-bin/), a putative full-length ORF of *PtrDFR2*, encoding 376 amino acids, was obtained. Based on this sequence information, specific primers were designed to amplify the full-length mRNAs that encode for *PtrDFR1* and *PtrDFR2* (see Materials and Methods for more details).

In a previous study, a cDNA clone (PtDFR) encoding dihydroflavonol-4-reductase, whose expression is induced by herbivory attack, was isolated from trembling aspen [7]. Sequence comparison revealed that PtDFR shared a much higher amino acid identity with PtrDFR1 (96.3%) than with PtrDFR2 (81.5%), indicating that the PtDFR gene may encode a DFR1 protein. *DFR* genes have also been cloned from other plant species, such as *A. thaliana* (*AtDFR*, GenBank Accession No. AAA32783), *M. truncatula* (*MtDFR1*, GenBank Accession No. AY389346) and *Oryza sativa* (*OsDFR*, GenBank Accession No. BAA36182). Amino acid sequence alignments showed that PtrDFR1 shared 71.4%, 71.3% and 62.0% identity with AtDFR, MtDFR1 and OsDFR, respectively (Figure 1B). The DFR enzyme catalyzes the NADPH-dependent reduction of 2*R*,3*R*-*trans*-dihydroflavonols to leucoanthocyanidins in the flavonoid biosynthetic pathway [37]. A putative NADP binding site (aa 10–30, VTGASGFIGSWLI/VMRLLEKGY) with very high sequence similarity with other DFRs [38] was also present in the same region (near the N-terminus) of the poplar DFR amino acid sequences (Figure 1B).

**Figure 2:**
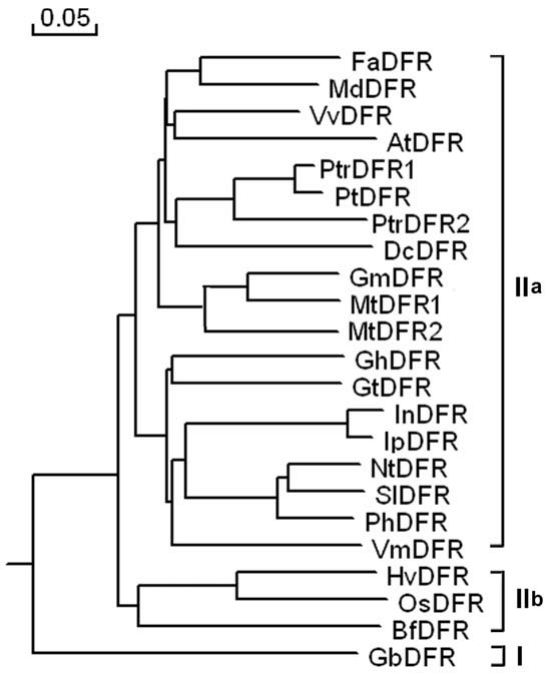
Phylogenetic relationships between PtrDFRs and other DFR proteins from other plant species. Phylogenetic and evolutionary analyses were performed using the neighbor-joining method by DNAMAN software. Additional sequences include *Medicago truncatula* (MtDFR1, AAR27014), *Populus tremuloides* (PtDFR, AY147903), *Arabidopsis thaliana* (AtDFR, AAA32783), *Fragaria x ananassa* (FaDFR, AAC25960), *Malus* x *domestica* (MdDFR, AAD26204), *Vitis vinifera* (VvDFR, CAA53578), *Dianthus caryophyllus* (DcDFR, CAA91924), *Gerbera hybrid* (GhDFR, CAA78930), *Gentiana triflora* (GtDFR, AA12736), *Ipomoea nil* (InDFR, BAA22072), *Ipomoea purpurea* (IpDFR, BAA74700), *Nicotiana tabacum* (NtDFR, ABN80437), *Solanum lycopersicum* (SiDFR, CAA79154), *Petunia x hybrida* (PhDFR, AAF60298), *Vaccinium macrocarpon* (VmDFR, AAL89714), *Hordeum vulgare* (HvDFR, AAB20555), *Bromheadia finlaysoniana* (BfDFR, AAB62873) and *Ginkgo biloba* (GbDFR, AAU95082).

To further investigate the sequence homology of PtrDFRs to other known DFRs, a rooted phylogenetic tree was constructed by using the predicated amino acid sequences from 20 species. As shown in Figure 2, these DFR proteins were clustered into two distinct groups. Only GbDFR from gymnosperm *Ginkgo biloba* belonged to Group I while other DFRs from angiosperm species belonged to Group II. In angiosperm DFRs, HvDFR (*Hordeum vulgare*), OsDFR (*O. sativa*) and BfDFR (*Bromheadia finlaysoniana*) from monocot plant species formed a subgroup (II-a) which was distinct from the dicot subgroup (II-b). These results are consistent with a recently published phylogenetic analysis of the DFR family [22].

### Characterization of expression patterns of *PtrDFR1* and *PtrDFR2*

**Figure 3:**
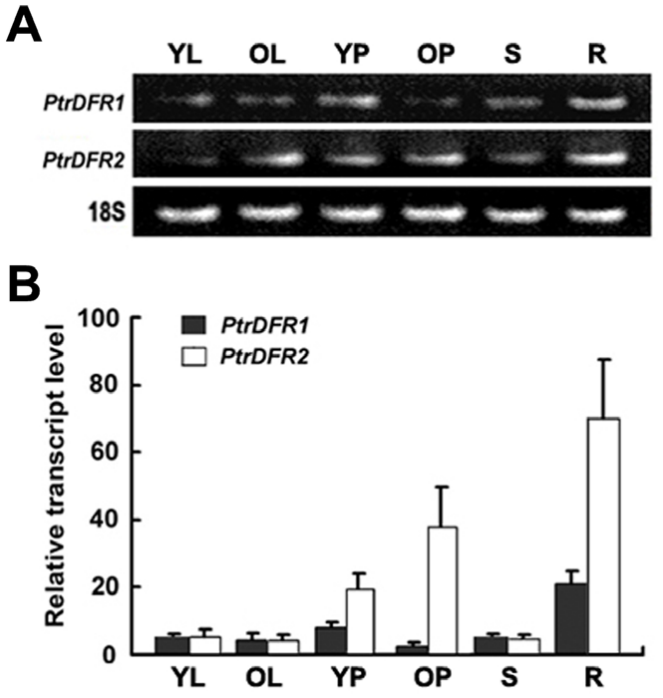
*PtrDFR1* and *PtrDFR2* transcript levels in different tissues of *P. trichocarpa*. (**A**) Gel analysis of semiquantitative reverse transcriptase (RT)-PCR with transcript-specific *DFR* primers. (**B**) Expression levels were determined by qRT-PCR. Values represent averages of three biological replicates, each with two technical replicates. *Actin* expression of poplar was used as a control. Total RNA was isolated from poplar tissues: root (R), shoot (S), young leaf (YL), old leaf (OL), young petiole (YP) and old petiole (OP).

In *M*. *truncatula*, transcript accumulation of both *DFR* genes was highest in young seeds and flowers, consistent with the accumulation of CTs and leucoanthocyanidins in these tissues [8]. It was established that the two *DFR* genes exhibited different expression patterns in *Populus*. *PtrDFR2* transcripts were detected in both roots and leaves while *PtrDFR1* expression was very weak in leaves [22]. In this study, the expression levels of the *PtrDFR* genes in various tissues were analyzed by quantitative real-time PCR by using gene-specific primers that can distinguish the two highly similar *PtrDFR* transcripts. Quantitative real-time PCR analysis showed that both *PtrDFR1* and *PtrDFR2* transcripts were found in each tested tissue, but were most concentrated in roots (Figure 3). *PtrDFR2* transcripts were more than twice as abundant as *PtrDFR1* in young petioles and were 15 times more abundant than in old petioles. In roots, the relative level of *PtrDFR2* transcripts was approximately three times higher than that of *PtrDFR1* transcripts. In contrast, *PtrDFR2* expression was relatively lower in stems compared to *PtrDFR1* expression. Neither *PtrDFR* gene was highly expressed in stems nor in young or mature leaves of poplar plants.

### Effect of ectopic expression of *PtrDFR1* and *PtrDFR2* on flower color in tobacco

Previous studies have shown that overexpression of the DFR genes from cranberry and *M. truncatula* in tobacco (*N. tabacum*) resulted in an increase in anthocyanin accumulation and a change in flower color [8], [39]–[41]. We found that the two *PtrDFR* genes exhibited distinct expression patterns in *Populus*, but it is not clear whether or not these enzymes can perform different functions in anthocyanin biosynthesis. To investigate the function of the PtrDFR proteins *in vivo*, transgenic tobacco plants that expressed *PtrDFR* genes under the control of the CaMV 35S promoter were produced. The pBI121 vector carrying the β-glucuronidase gene driven by the CaMV 35S promoter [42] was used as a control for comparison (named pBI121 control vector). PCR analysis using gene-specific primers with genomic DNA from leaf samples of putative transgenic plants identified the integration of *nptII*, *PtrDFR1* and *PtrDFR2* in the tobacco genome (Figure S1).

**Figure 4:**
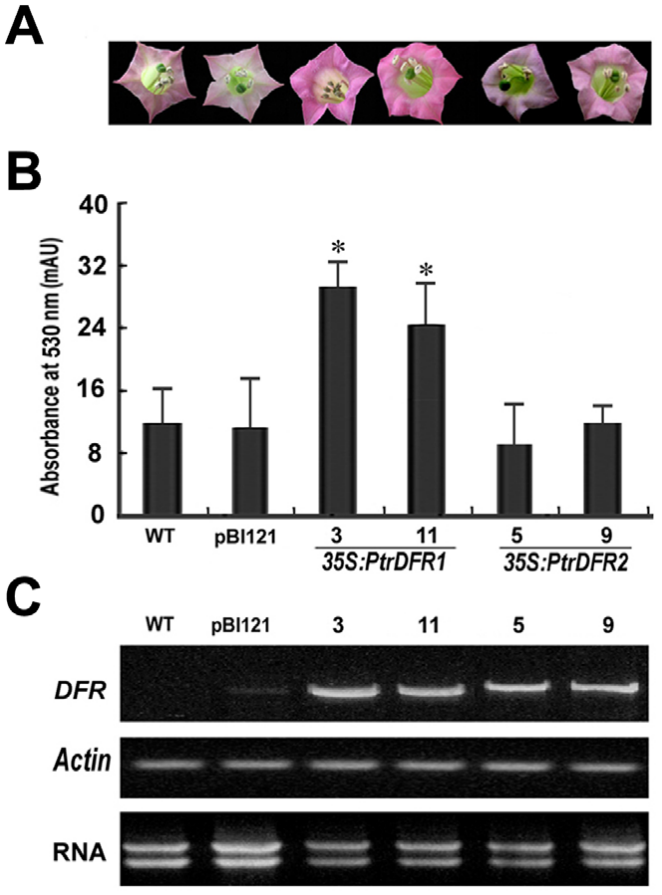
Effect of *PtrDFR1* and *PtrDFR2 in vivo* on anthocyanin accumulation in transgenic tobacco flowers. (**A**) Overexpression of *PtrDFR1* resulted in a visible increase in anthocyanin accumulation in the corolla of transgenic tobacco flowers (lines 3 and 11), relative to untransformed lines (WT) and pBI121 transgenic control (pBI121) or the *35S:PtrDFR2* transgenics (5 and 9). (**B**) Quantitation of anthocyanin levels in transgenic tobacco flowers with a spectrophotometer. Error bars are SDs from three independent experiments. Both the lines harboring the *35S:PtrDFR1* gene had significantly higher anthocyanin levels compared with wild-type and the pBI121 transgenic control (based on a Student's *t* test analysis limit of *P*≤0.05). No lines overexpressing the *35S:PtrDFR2* gene showed significant increases in anthocyanins. (**C**) RT-PCR analysis of *PtrDFR1* and *PtrDFR2* expression in transgenic tobacco plants.

In general, flowers of the wild-type control tobacco plants (*N. tabacum cv* Xanthi) that were used for transformation exhibited white or pale pink colors under greenhouse conditions. Transgenic plants harboring the *35S:PtrDFR1* transcription cassette produced much darker pink flowers than were observed on wild-type control plants or transgenic plants carrying either the *35S:PtrDFR2* transcription cassette or the pBI121 control vector (Figure 4A). Further, anthocyanin pigments were extracted from the corollas of flowers of various plants and roughly measured spectrophotometrically. Compared to either wild-type control or pBI121 control vector transgenic plants, a significantly higher accumulation of anthocyanins was detected in the *PtrDFR1* overexpression transgenic plants (Figure 4B). None of the *PtrDFR2* transgenic plants showed such a significant increase. RT-PCR analyses proved overexpression of *PtrDFR1* or *PtrDFR2* in each correlated transgenic tobacco line (Figure 4C). Together, these results suggested that the PtrDFR1 protein may interact with the endogenous enzymes of anthocyanin biosynthetic pathways in tobacco and result in the increase of anthocyanin accumulation *in vivo*, whereas the PtrDFR2 protein does not seem to have such a function. Our findings are consistent with a previous report on *M. truncatula* [8] in which overexpression of *MtDFR1* in transgenic tobacco resulted in a visible increase in anthocyanin accumulation in flowers, while *MtDFR2* did not, indicating that unexpected properties and differences are found in two DFR proteins of the same orthology.

### Functional characterization of *PtrDFR* genes in CT biosynthesis in transgenic poplar

It has been previously demonstrated that CT concentrations are as high as 50% in cottonwood (*P. angustifolia*) [43], [44] and constitute up to 18% more of leaf dry weight than in aspen (*P. tremuloides*) [45]. CT accumulation can be induced by herbivores and pathogenic fungi in woody plants [46], [7], indicating that CTs play important roles in defense and protection against biotic stresses [47]. To date, the biosynthetic pathways for CTs and a large number of polyphenolic compounds have been well established [7], [22]. *DFR* encodes a key enzyme in the later steps of CT synthesis in plants, and overexpression of a *DFR* gene in the forage legume *Lotus corniculatus* resulted in an alteration of CT levels [48], [49]. To further determine the function of *PtrDFR*s in the CT biosynthetic pathway, their ORFs, flanked by the 35S promoter, were introduced into *P. tomentosa* Carr. More than 20 independent kanamycin-resistant transgenic lines harboring either p*35S:PtrDFR1* or p*35S:PtrDFR2* expression cassettes were produced. All transgenic plants were confirmed with PCR analysis by using gene-specific primers (Figure S2). No obvious morphological differences were observed between the transgenic and wild-type poplar plants. Three representative lines of each construction were selected for further analysis.

**Figure 5:**
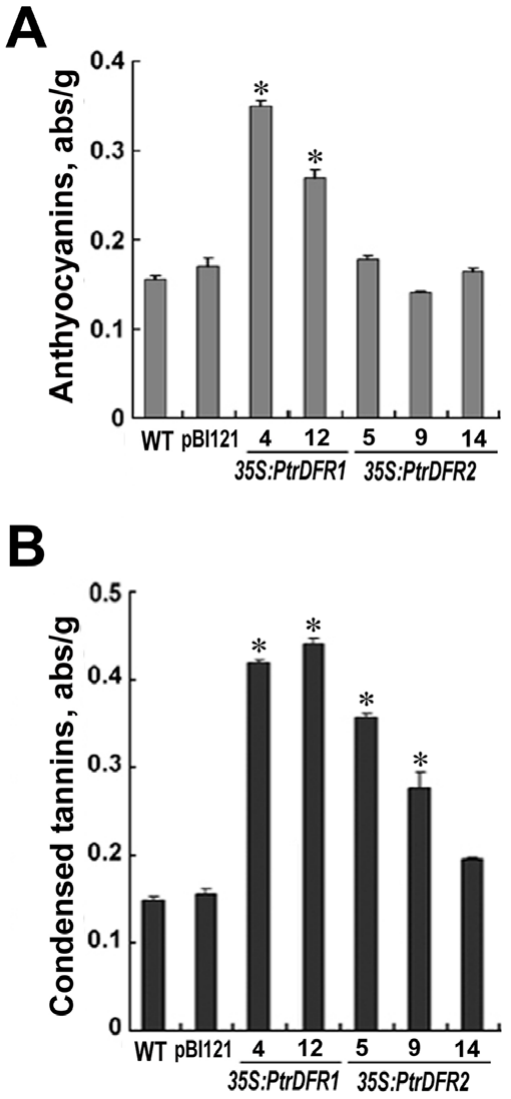
Anthocyanin and condensed tannin levels in *PtrDFR1* and *PtrDFR2* transgenic *P. tomentosa* Carr. (**A**) The estimated relative anthocyanin contents at 530 nm absorbance. (**B**) The estimated relative condensed tannin contents at 500 nm absorbance. Error bars represent SDs from three independent experiments. Asterisks indicate a statistically significant difference between wild-type and transgenic plants (P≤0.05 by Student's *t*-test).

Spectrophotometric quantification of total CTs showed that all transgenic lines that carried the *35S*:*PtrDFR1* or *35S*:*PtrDFR2* transcription cassettes exhibited an increase in CT content in leaf tissues compared to wild-type plants and the transgenic controls of the pBI121 control vector (Figure 5B). In *35S:PtrDFR1* lines 4 and 12, a three-fold increase in the CT amount was detected in leaf tissues when compared to the wild-type control. Similarly, in *35S:PtrDFR2* lines, the concentration of total CTs in lines 5 and 9 was more than twice as high as those in the wild-type control and pBI121 transgenic control (Figure 5B). In addition, total anthocyanin levels were also measured spectrophotometrically. A dramatic increase in anthocyanin accumulation was detected in *PtrDFR1* overexpression transgenic poplar plants, whereas none of the *PtrDFR2* transgenic lines showed a significant increase of anthocyanins (Figure 5A). These results are consistent with the findings in transgenic tobacco as described above.

**Figure 6:**
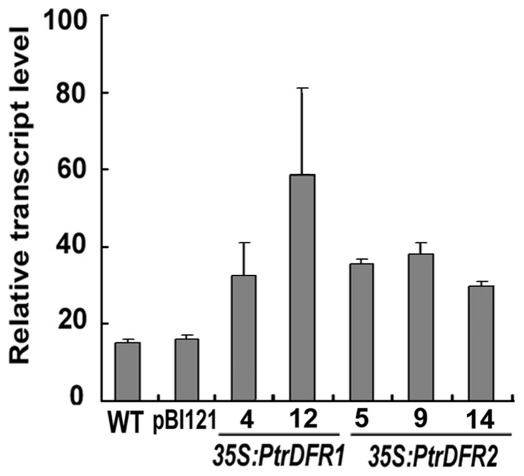
Determination of transcript levels of *PtrDFR1* and *PtrDFR2* in transgenic poplar plants by quantitative real-time PCR. The *PtrDFR1* expression was significantly increased in transgenic lines 4 and 12, whereas the *PtrDFR2* transcript level was higher in all three transgenic lines than in wild-type and pBI121 transgenic controls.

Quantitative real-time PCR analysis confirmed that much higher levels of *PtrDFR1* transcripts accumulated in *35S:PtrDFR1* transgenic lines (e.g., 4 and 12) compared to the wild-type and pBI121 transgenic control lines (Figure 6). Similarly, all *35S:PtrDFR2* lines accumulated high levels of *PtrDFR2* transcripts. These results are in agreement with the previous analysis of flavonoid accumulation in the leaf tissues of *35S:PtrDFR1* lines. However, enhanced transcription levels of *PtrDFR2* were not accompanied by a significant increase in the amount of total anthocyanins. Obviously, *PtrDFR2* transcription levels correlate poorly with anthocyanin accumulation in *Populus*, but correlate well with CT accumulation. Taken together, these results suggest that the two *DFR* genes in *P*. *trichocarpa* may be specialized for anthocyanin synthesis or CT pathways due to gene duplication or pathway redundancy during plant evolution. Similarly, multiple *DFR* genes are present in *L. japonicus* and *M. truncatula* genomes, and these DFR proteins possess different catalytic activities in plants, suggesting a single duplication event followed by functional divergence or two independent duplication events followed by independent sub- or neo-functionalization events [8], [50]. To further determine their functions in the flavonoid pathway, detailed biochemical characterization of purified PtrDFR isozymes will be performed in the future.

## Supporting Information

Figure S1 **PCR analysis of transgenic tobacco plants.** (**A**) PCR amplification using primers specific for the production of a 741-bp *NPTII* fragment. (**B**) PCR amplification using primers specific for the production of a 1,375-bp *PtrDFR1* fragment. (**C**) PCR amplification using primers specific for the production of a 1,128-bp *PtrDFR2* fragment. M, D2000 DNA Ladder; WT, wild-type plants; pBI121, transgenic control; Plasmid, corresponding plasmid DNA (positive control); Lanes 1–16, independent transgenic lines. Numbers on the left indicate DNA marker sizes in base pairs.
(TIF)

Figure S2 **PCR analysis of transgenic** ***P. tomentosa*** **Carr. plants.** PCR amplification using primers designed for a 741-bp fragment of the *NPTII* gene using total genomic DNA as the template. (**A**) Of *35S:PtrDFR1* transgenic lines (2, 3, 4, 5, 6, 7, 8, 10, 11, 12 and 15). (**B**) Of *35S:PtrDFR2* transgenic lines (1, 2, 4, 5, 7, 8, 9, 10, 11 and 14). M, D2000 DNA Ladder; WT, wild-type plants; pBI121, transgenic control; Plasmid, corresponding plasmid DNA (positive control). Numbers on the left indicate DNA marker sizes in base pairs.
(TIF)
