# Supplementary figures and images for: Molecular Cloning and Characterization of Two Genes Encoding Dihydroflavonol-4-Reductase from Populus trichocarpa
Source: PLoS One. 2012 Feb 17;7(2):e30364. doi: 10.1371/journal.pone.0030364 (PMC3281835; doi:10.1371/journal.pone.0030364)

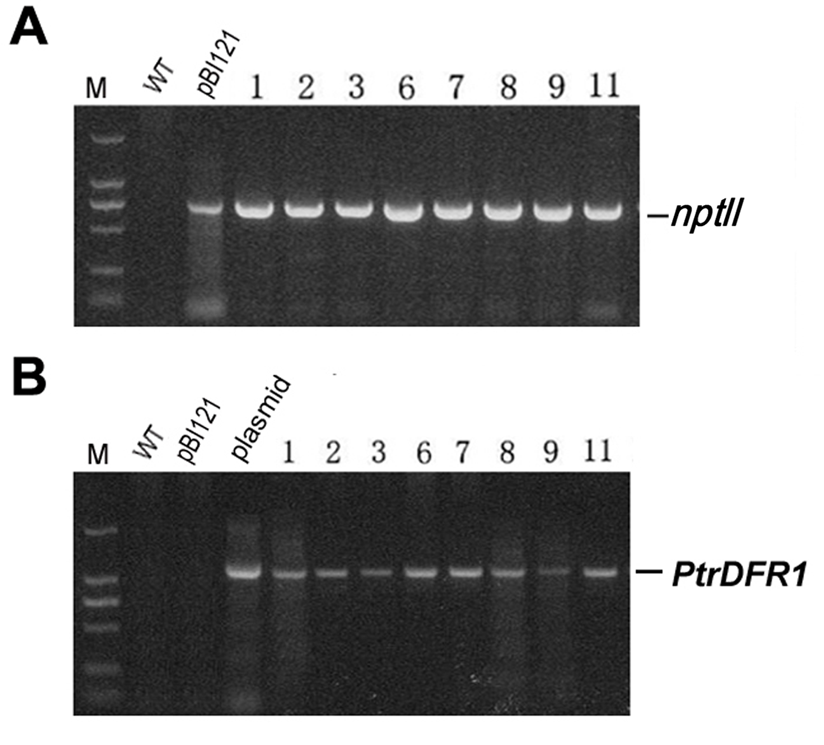

Supplement: Figure S1 — PCR analysis of transgenic tobacco plants. (A) PCR amplification using primers specific for the production of a 741-bp NPTII fragment. (B) PCR amplification using primers specific for the production of a 1,375-bp PtrDFR1 fragment. (C) PCR amplification using primers specific for the production of a 1,128-bp PtrDFR2 fragment. M, D2000 DNA Ladder; WT, wild-type plants; pBI121, transgenic control; Plasmid, corresponding plasmid DNA (positive control); Lanes 1–16, independent transgenic lines. Numbers on the left indicate DNA marker sizes in base pairs. (TIF) [file pone.0030364.s001.tif]

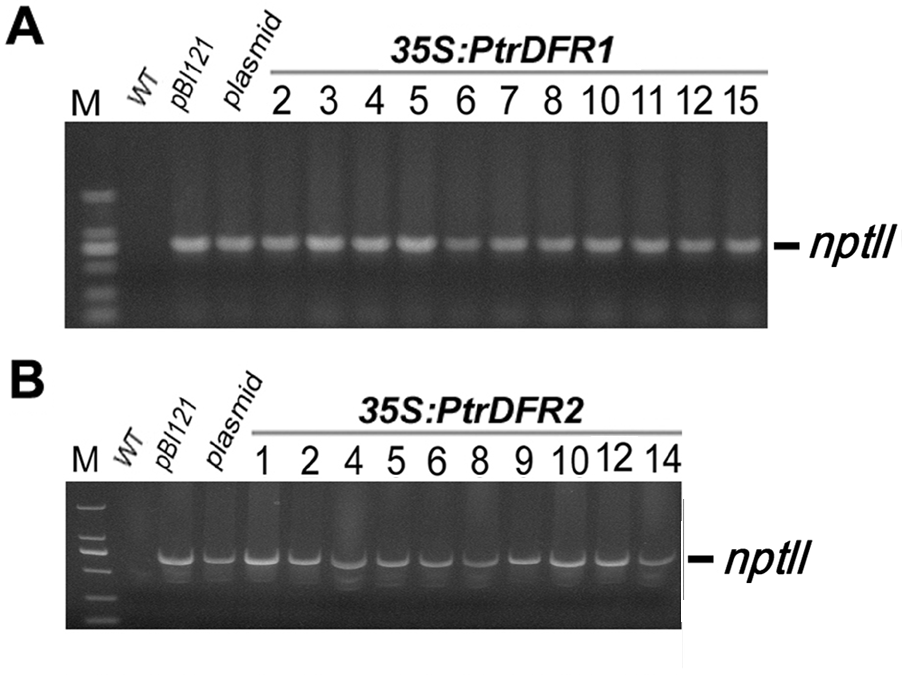

Supplement: Figure S2 — PCR analysis of transgenic P. tomentosa Carr. plants. PCR amplification using primers designed for a 741-bp fragment of the NPTII gene using total genomic DNA as the template. (A) Of 35S:PtrDFR1 transgenic lines (2, 3, 4, 5, 6, 7, 8, 10, 11, 12 and 15). (B) Of 35S:PtrDFR2 transgenic lines (1, 2, 4, 5, 7, 8, 9, 10, 11 and 14). M, D2000 DNA Ladder; WT, wild-type plants; pBI121, transgenic control; Plasmid, corresponding plasmid DNA (positive control). Numbers on the left indicate DNA marker sizes in base pairs. (TIF) [file pone.0030364.s002.tif]
